# Downregulation of protease activated receptor expression and cytokine production in P815 cells by RNA interference

**DOI:** 10.1186/1471-2121-10-62

**Published:** 2009-09-07

**Authors:** Liya Qiao, Huiyun Zhang, Shandong Wu, Shaoheng He

**Affiliations:** 1 Allergy and Inflammation Research Institute, Shantou University Medical College, 22 Xin-ling Road, Shantou, Guangdong 515041, PR China; 2 Zhejiang University Medical College, Science and Research Building, Block C, Hangzhou 310013, PR China; 3 Clinical Research Center, the First Affiliated Hospital of Nanjing Medical University, Nanjing 210029, Jiangsu Province, PR China

## Abstract

**Background:**

Protease-activated receptors (PAR) are seven transmembrane G-coupled receptors comprising four genes (PAR-1 ~ PAR-4). Mast cell has been identified to be able to express PARs and release an array of cytokines upon activation. Recently, it was reported that interleukin (IL)-12 could regulate the expression of PARs in mast cells, and tryptase could induce IL-4 and IL-6 release from mast cells. In order to further investigate the issues, RNA interference (RNAi) technique was employed and small interfering RNAs (siRNA) of PARs were transfected in P815 cells.

**Results:**

The results showed that siRNAs for PAR-1, PAR-2 and PAR-4 significantly downregulated expression of PAR-1, PAR-2 and PAR-4 mRNAs and proteins in P815 cells at 24, 48 and 72 h following transfection. siRNA PAR-1.2 and siRNA PAR-4.2 significantly reduced IL-12 induced upregulation of PAR-1 and PAR-4 expression, respectively when P815 cells were transfected with them for 48 h. siRNA PAR-2.3 blocked IL-12 induced downregulation of PAR-2 expression on both mRNA and protein levels. It was also observed that siRNA PAR-2.3 and siRNA PAR-1.2 reduced trypsin induced IL-4 release by approximately 92.6% and 65.3%, and SLIGKV-NH_2 _induced IL-4 release by 82.1% and 60.1%, respectively. Similarly, siRNA PAR-2.3 eliminated tryptase-induced IL-4 release by 75.3%, and siRNA PAR-1.2 diminished SFLLR-NH_2 _induced IL-4 release by 79.3%. However, siRNA PAR-1.2, siRNA PAR-2.3 and siRNA PAR-4.3 at 10 nM did not show any effect on tryptase-induced IL-6 release from P815 cells.

**Conclusion:**

In conclusion, siRNAs of PARs can modulate PAR expression and PAR related cytokine production in mast cells, confirming that PARs are likely to play a role in allergic reactions.

## Background

As the primary effector cell, mast cell is actively involved in the pathogenesis of both acute and chronic allergic diseases[1,2]. Upon activation, mast cell can release not only its preformed but also newly generated mediators to fulfill its biological functions. Besides histamine, heparin and proteases, mast cells can synthesize and secrete a variety of cytokines[2] such as IL-4, IL-5 and IL-6. These cytokines have been well documented for their ability to regulate cell behavior including growth, secretion and migration in physiological and pathological conditions.

In recent years, PARs have been indentified as receptors for serine proteinases. PARs are a subfamily of seven transmembrane G-protein-coupled receptors. Among them, PAR-1, PAR-3 and PAR-4 serve as a receptor of thrombin[3-5]; PAR-1, PAR-2 and PAR-4 are receptors of trypsin and PAR-2 is a receptor of tryptase. These serine proteinases have been discovered to play a crucial role in allergic inflammation. Tryptase was reported to be able to stimulate microvascular leakage in the skin of guinea pigs[6], to induce inflammatory cell accumulation in the peritoneum of mice[7], to elicit histamine release from mast cells^6 ^and to enhance monocyte chemoattractant protein-1 (MCP-1) and IL-8 production in human endothelial cells[8]. Trypsin was found to be able to induce histamine release from human tonsil and skin mast cells[9] and to stimulate IL-8 and IL-6 release from human respiratory epithelial cells[10].

RNAi is a process in which double-stranded RNA (dsRNA) induces the posttranscriptional degradation of homologous transcripts. It has been observed in variety of organisms including plants, fungi, insects, protozans and mammals[11,12]. RNAi can be initiated by exposing cells to dsRNA either via transfection or endogenous expression. dsRNAs are processed into 21- to 23-nt double-stranded fragments known as siRNAs[12]. With unprecedented speed, RNAi has advanced from its basic discovery in lower organisms to becoming a powerful genetic tool in mammals. The aim of this study is to investigate the effect of PAR-1, PAR-2 and PAR-4 gene silencing on the expression of PARs and cytokine production in P815 cells by using RNAi.

## Results

### Reduction of PAR-1 expression in P815 cells by siRNA

**Figure 1:**
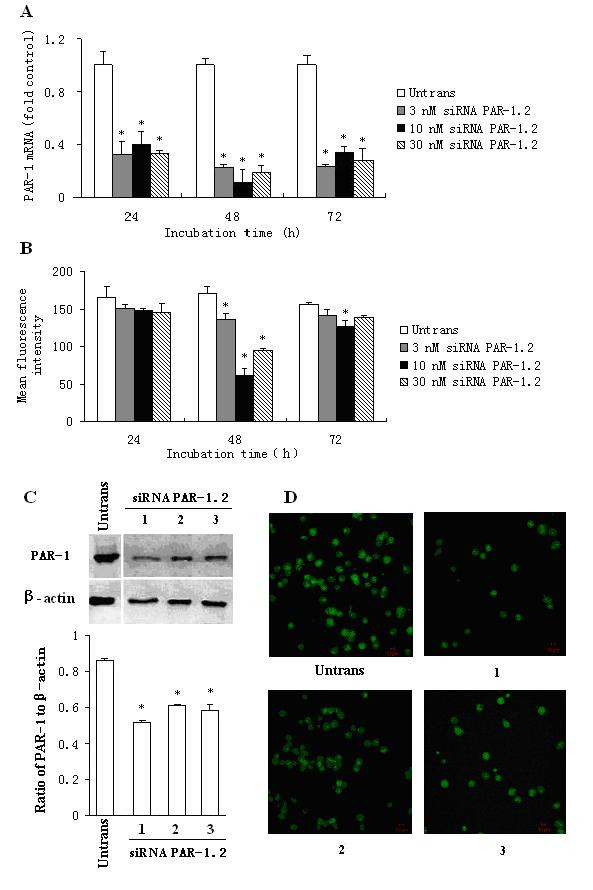
**Effect of siRNA PAR-1.2 on PAR-1 expression in P815 cells**. P815 cells were transfected with siRNA PAR-1.2 for 24, 48 and 72 h. The PAR-1 expression was determined by real-time quantitative PCR (A), flow cytometry (B), Western blot (C) and immunofluorescent analysis (D). In (C) and (D), 1 = 3 nM, 2 = 10 nM and 3 = 30 nM of siRNA PAR-1.2. Values shown are mean ± SEM for four to five independent experiments. * P < 0.05 compared with the untransfected control.

Three target sequences in the coding region of PAR-1 mRNA were selected as siRNA sequences, namely siRNA PAR-1.1, siRNA PAR-1.2 and siRNA PAR-1.3. Among the 3 siRNA PAR-1s, siRNA PAR-1.2 appeared most effective in inhibition of PAR-1 mRNA expression in P815 cells (data not shown). Approximately up to 80.5% reduction of PAR-1 mRNA expression was observed when P815 cells were transfected with 10 nM of siRNA PAR-1.2 for 48 h. At 24 or 72 h following transfection, however siRNA PAR-1.2 showed less degree of inhibition of PAR-1 mRNA expression (Figure 1A). Flow cytometry analysis revealed that PAR-1 protein expression was eliminated by up to 60.1% when P815 cells were transfected with 10 nM of siRNA PAR-1.2 for 48 h (Figure 1B). Because siRNA PAR-1.2 showed greater inhibitory effect on PAR-1 expression at 48 h than at 24 or 72 h following incubation, the 48 h incubation period was chosen as the fixed time point for the following Western blot and immunofluorescent analysis. Approximately up to 46.3% and 42.6% inhibition of PAR-1 expression was observed by Western blot (Figure 1C) and immunofluorescent analysis (Figure 1D), respectively. siRNA PAR-1.1 and siRNA PAR-1.3 also showed significant inhibition of PAR-1 protein expression in P815 cells (data not shown).

### Reduction of PAR-2 expression in P815 cells by siRNA

**Figure 2:**
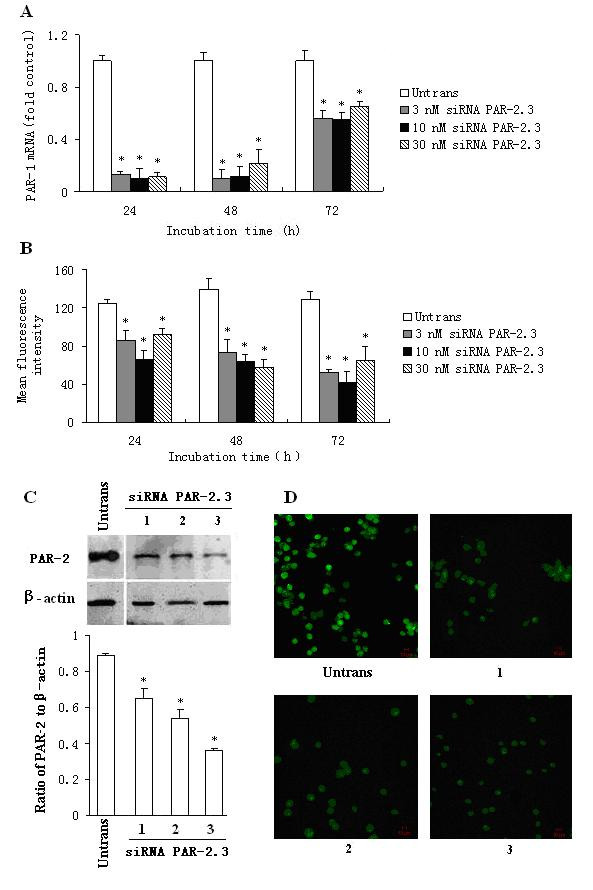
**Effect of siRNA PAR-2.3 on PAR-2 expression in P815 cells**. P815 cells were transfected with siRNA PAR-2.3 for 24, 48 and 72 h. The PAR-2 expression was determined by real-time quantitative PCR (A), flow cytometry (B), Western blot (C) and immunofluorescent analysis (D). In (C) and (D), 1 = 3 nM, 2 = 10 nM and 3 = 30 nM of siRNA PAR-2.3. Values shown are mean ± SEM for four to five independent experiments. * P < 0.05 compared with the untransfected control.

Of the three siRNA PAR-2s, siRNA PAR-2.1 and PAR-2.3, but not PAR-2.2 downregulated PAR-2 mRNA expression by up to 69.3% and 82.1% at 48 h following transfection (Figure 2A). Therefore siRNA PAR-2.3 was employed as a representative throughout the study. siRNA PAR-2.3 also suppressed PAR-2 protein expression during flow cytometry, Western blot and immunofluorescence staining analysis by up to 56.8% (Figure 2B), 60.2% (Figure 2C) and 59.3% (Figure 2D), respectively following 48 h incubation period. At 24 and 72 h following incubation, siRNA PAR-2.3 showed significant downregulation of PAR-2 expression on both mRNA and protein levels.

### Reduction of PAR-4 expression in P815 cells by siRNA

**Figure 3:**
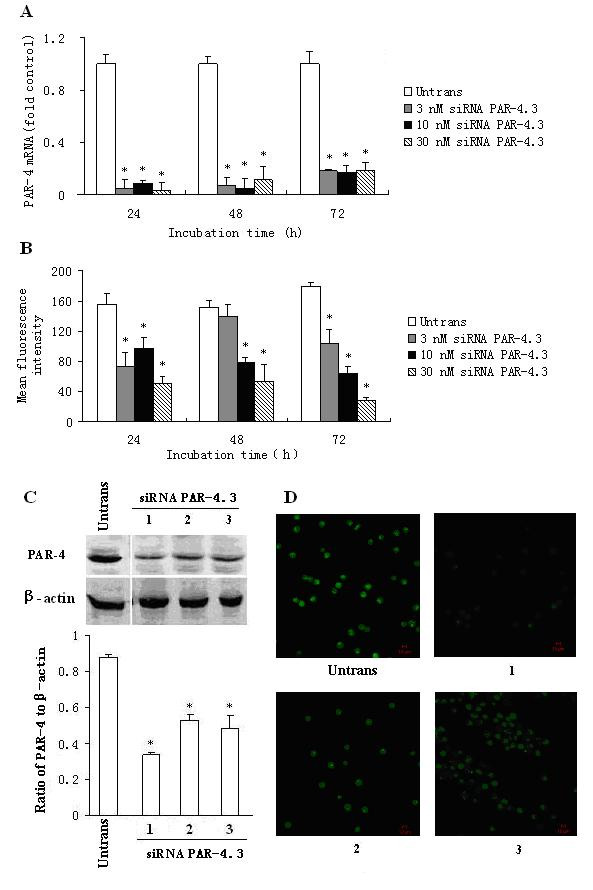
**Effect of siRNA PAR-4.3 on PAR-4 expression in P815 cells**. P815 cells were transfected with siRNA PAR-4.3 for 24, 48 and 72 h. The PAR-4 expression was determined by real-time quantitative PCR (A), flow cytometry (B), Western blot (C) and immunofluorescent analysis (D). In (C) and (D), 1 = 3 nM, 2 = 10 nM and 3 = 30 nM of siRNA PAR-4.3. Values shown are mean ± SEM for four to five independent experiments. * P < 0.05 compared with the untransfected control.

All three siRNAs of PAR-4, namely siRNA PAR-4.1, siRNA PAR-4.2 and siRNA PAR-4.3 were able to downregulate the PAR-4 expression on both mRNA and protein levels in P815 cells (data not shown). However, siRNA PAR-4.3 showed the most potent effect on P815 cells among the 3 siRNAs. It was observed that siRNA PAR-4.3 suppressed approximately up to 91.2% PAR-4 mRNA expression at 24 and 48 h following transfection (Figure 3A). siRNA PAR-4.3 inhibited also up to 82.3% PAR-4 protein expression on flow cytometry analysis at 72 h (Figure 3B). Similarly, siRNA PAR-4.3 eliminated PAR-4 expression by 52.3 and 91.3% on Western blot (Figure 3C) and immunofluorescence staining analysis (Figure 3D) in P815 cells at 48 h following transfection.

### Inhibition of IL-12 induced changes in PAR expression in P815 cells by siRNA

**Figure 4:**
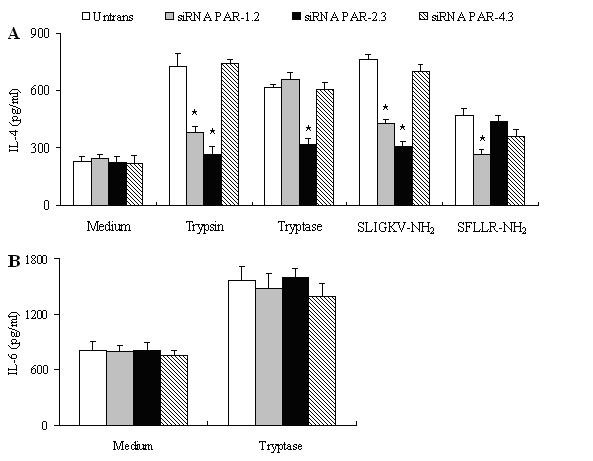
**Effect of siRNA PARs on IL-12 (10 ng/ml) induced expression of PARs in P815 cells**. Cells were transfected with 10 nM of siRNA PAR-1.2, siRNA PAR-2.3 or siRNA PAR-4.3 for 48 h before IL-12 being added for 6 h at 37°C. The PAR-1 (A), PAR-2 (B) and PAR-4 (C) mRNA expression was determined by real-time quantitative PCR. PAR-2 (D) and PAR-4 (E) protein expression was analyzed by flow cytometry analysis and expressed as mean fluorescence intensity. Values shown are mean ± SEM for four to five independent experiments. * P < 0.05 compared with the response of untransfected cells to the corresponding stimulus.

IL-12 at the concentration of 10 ng/ml induced approximately 2.4 and 3.0 fold upregulation of expression of PAR-1 (Figure 4A) and PAR-4 mRNAs (Figure 4C) in P815 cells, respectively at 6 h following incubation. In contrast, 10 ng/ml of IL-12 downregulated PAR-2 mRNA expression in P815 cells by approximately up to 40.1% (Figure 4B). It was observed that IL-12 induced upregulation of PAR-4 (Figure 4E), but downregulation of PAR-2 protein expression (Figure 4D). siRNA PAR-1.2 at 10 nM and siRNA PAR-4.2 at 10 nM significantly reduced IL-12 induced upregulation of PAR-1 (Figure 4A) and PAR-4 expression (Figure 4C, Fig. 4E), respectively when P815 cells were transfected with them for 48 h. siRNA PAR-2.3 at 10 nM was able to block IL-12 induced downregulation of PAR-2 expression on both mRNA (Figure 4B) and protein (Figure 4D) levels in P815 cells.

### Inhibition of protease-induced cytokine release by siRNA

**Figure 5:**
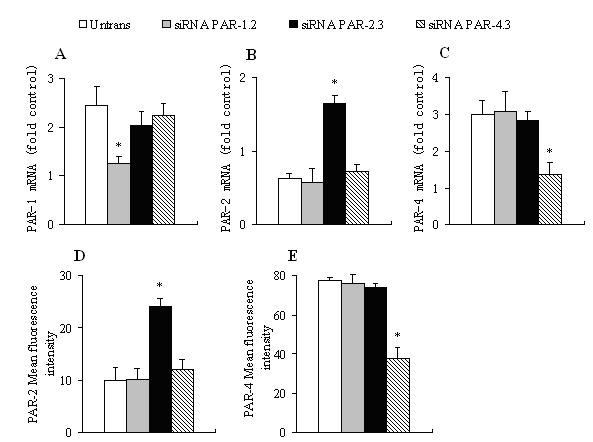
**Effect of siRNA PARs on IL-4 (A) and IL-6 (B) release from P815 cells**. Cells were transfected with 10 nM of siRNA PAR-1.2, siRNA PAR-2.3 or siRNA PAR-4.3 for 48 h before protease or agonist of PAR being added for 16 h at 37°C. Values shown are mean ± SEM for four to five independent experiments. * P < 0.05 compared with the response of untransfected cells to the corresponding stimulus.

Tryptase at 1 μg/ml, trypsin at 100 ng/ml, SFLLR-NH_2 _at 100 μM and SLIGKV-NH_2 _at 100 μM induced 3.0, 2.4, 3.2 and 1.7 fold increase in IL-4 release from P815 cells, respectively at 16 h following incubation (Figure 5A). However, thrombin at 3 U/ml, RLLFS-NH_2 _at 100 μM, VKGILS-NH_2 _at 100 μM, GYPGQV-NH_2 _at 100 μM and VQGPYG-NH_2 _at 100 μM failed to elicit IL-4 release from P815 cells following 16 h incubation period (data not shown). Trypsin induced IL-4 release was almost completely abolished by 10 nM of siRNA PAR-2.3, and reduced approximately 65.3% by 10 nM of siRNA PAR-1.2. Similarly, SLIGKV-NH_2 _induced IL-4 release was inhibited by 82.1% and 60.1% by 10 nM of siRNA PAR-2.3 and siRNA PAR-1.2, respectively. It was observed that tryptase- induced IL-4 release was eliminated by approximately 75.3% by 10 nM of siRNA PAR-2.3, and SFLLR-NH_2 _induced IL-4 release was diminished by approximately 79.3% by 10 nM of siRNA PAR-1.2 (Figure 5A).

Tryptase at 1 μg/ml, but not thrombin at 3 U/ml, trypsin at 100 ng/ml, SFLLR-NH_2 _at 100 μM, SLIGKV-NH_2 _at 100 μM and GYPGQV-NH_2 _at 100 μM was able to provoke 2.1 fold increase in IL-6 secretion from P815 cells at 16 h following incubation. However, siRNA PAR-1.2, siRNA PAR-2.3 and siRNA PAR-4.3 at 10 nM did not show any effect on tryptase-induced IL-6 release from P815 cells (Figure 5B).

## Discussion

Delivery of synthetic siRNA or vector-based siRNA expression systems to target cells can reverse the expression and function of the gene under consideration. In recent years, siRNA technique has been widely used as one of the most powerful tools for investigation of protein functions in mammalian cells [12]. Although P815 cell is a mouse mastocytoma cell line, rather than primary mast cells, it has been shown to express PAR-1, PAR-2, PAR-3 and PAR-4 genes and proteins[13]. To confirm these findings, we examined the effect of siRNAs on PAR expression in the present study. Our data showed, for the first time, that siRNAs of PAR-1, PAR-2 and PAR-4 are able to reduce PAR-1, PAR-2 and PAR-4 expression to a great extent, indicating these small RNAs disrupt the normal PAR generation process of P815 cells. As for most dsRNAs, siRNAs of PAR-1, PAR-2 and PAR-4 fail to completely block PAR production in P815 cells. Incomplete siRNA-induced gene suppression may result from the presence of a fraction of mRNA in a protected compartment such as spliceosomes, other nuclear locations or non-transfected cells[14]. Another study found that the activity of siRNA in mammalian cells is related to structural target accessibility[15].

PAR-1, PAR-2 and PAR-4 gene silencing can block IL-12 induced alteration of expression of PAR mRNAs and proteins in P815 cells, confirming that IL-12 is an effective modulator of PAR expression in mast cells. To our knowledge, this is the first work demonstrates the effects of PAR siRNAs on cytokine induced alteration of PAR expression. IL-12 regulates Th1 cell differentiation, while suppressing the expansion of Th2 cell clones[16]. It has been implicated in the pathogenesis of allergy[17]. Since mast cell has long been recognized as the primary effector cell of allergy and upregulation of PAR-2 expression was found in the airways of asthma[18], we anticipate that IL-12 is likely to be involved in the pathogenesis of asthma through its ability of regulation of PAR expression on mast cells.

We have previously showed that tryptase and trypsin can induce IL-4 release from P815 cells via a PAR-2 dependent mechanism[13]. Using siRNA technique in the present study, it is confirmed that trypsin provoked IL-4 release is mainly through PAR-2 and partially via PAR-1 related mechanisms, whereas tryptase elicited IL-4 release is dependent on activation of PAR-2. In contrast, tryptase induced IL-6 release is independent on the activation of PARs as siRNAs of PAR-1, PAR-2 and PAR-4 did not show any influence on the event. Since tryptase is a unique secretory product of mast cells and IL-4 is a classic Th2 cytokine which is actively involved in the pathogenesis of allergic reactions, induction of IL-4 release from mast cells by tryptase may suggest a self-amplification mechanism of allergic reactions. Inhibition of PAR dependent cytokine release by siRNAs of PARs has been reported before. Thus, thrombin induced IL-8 and VEGF release from prostate cancer cells[19] and IL-6 production from synovial fibroblasts[20] was blocked by siRNA of PAR-1, trypsin induced IL-8 production from human gastric epithelial cells (MKN45 cells) was inhibited by siRNA of PAR-2[21]. PAR independent release of IL-6 from P815 cells induced by tryptase was unexpected. However, a study demonstrated that beta-tryptase regulates IL-8 expression in airway smooth muscle cells by a PAR-2-independent mechanism[22] may help to explain our above observation.

## Conclusion

In conclusion, siRNAs of PAR-1, PAR-2, PAR-4 not only block their corresponding PAR expression, inhibit IL-12 induced alteration of expression of PARs, but also reduce tryptase and trypsin provoked IL-4 release from P815 cells, confirming that mast cell PARs are likely to be involved in the pathogenesis of allergic reactions. However, a further study using primary human mast cells would be better for evaluation the role of mast cell PARs in allergic reactions.

## Methods

### Reagents

The mouse mastocytoma cell line P815 was obtained from the American Type Culture Collection (Manassas, VA, USA). Cells culture reagents including Dulbecco's modified Eagle's medium (DMEM) and fetal bovine serum (FBS) were from HyClone (Logan, UT, USA). siPORT™ *NeoFX*™ Transfection Agent was from Ambion (Huntingdon, UK). TRIZOL Reagent was from Invitrogen (Carlsbad, CA, USA). ExScriptTM RT reagent kit and SYBR^® ^Premix Ex Taq TM (perfect real time) was from TaKaRa (TaKaRa Biotechnology Co. Ltd. DaLian, China). Primers for mouse PAR-1, PAR-2 and PAR-4 were synthesized by Invitrogen Biotechnology Co. Ltd, (Nanjing, China). Rabbit anti-mouse PAR-1, PAR-2, PAR-4 and rabbit anti-mouse β-actin monoclonal antibody were purchased from Santa Cruz Biotechnology (Santa Cruz, CA, USA). FITC-conjugated goat anti-rabbit polyclonal antibody was from BD Pharmingen (San Jose, CA, USA). Paraformaldehyde, bovine serum albumin (BSA, fraction V), trypsin, thrombin and peroxidase conjugated goat anti-rabbit immunoglobulins were from Sigma Inc. (St. Louis, MO, USA). Recombinant human lung β-tryptase was from Promega (Madison, WI, USA). Agonist peptides of PARs, as well as their reverse forms were synthesized in CL Bio-Scientific Inc (XiAn, China). The sequences of the active and reverse peptides were: PAR-1, SFLLR-NH_2 _and RLLFS-NH_2_; PAR-2, SLIGKV-NH_2 _and VKGILS-NH_2_; PAR-4, GYPGQV-NH_2 _and VQGPYG-NH_2_. Mouse IL-4 and IL-6 ELISA kits were from Pierce Biotechnology Inc. (Rockford, IL, USA). Most of other reagents such as salt and buffer components were analytical grade and obtained from Sigma.

### Small interfering RNA synthesis

The potential siRNAs of PAR-1, PAR-2 and PAR-4 were designed by using the prediction of single strand domains in the secondary mRNA structure and subsequent negative BLAST analyses, 3 sequences for each PAR (Table 1). A scrambled sequence was used as negative control, and β-actin siRNA was employed as positive control. No significant matching in mouse transcripts was found in BLAST analyses. siRNA were prepared by *in vitro *transcription using the siPORT™ *NeoFX*™ Transfection Agent, a lipid-based agent for reverse transfection.

**Table 1:** siRNA sequences of PARs

**Sequence**	**Sense strand (5'-3')**	**Antisense strand (5'-3')**
PAR-1.1	GGGUAGGGCAGUCUACUUAtt	UAAGUAGACUGCCCUACCCtc
PAR-1.2	GGUUCCAUGAGAAAAGGUUtt	AACCUUUUCUCAUGGAACCtt
PAR-1.3	CCAAGUGUAUUUCACAUAAtt	UUAUGUGAAAUACACUUGGtc
PAR-2.1	GCUGUACCUGAGGAUGUCAtt	UGACAUCCUCAGGUACAGCtc
PAR-2.2	GCAUCAGUAUCAGAAACUGtt	CAGUUUCUGAUACUGAUGCtt
PAR-2.3	CCAACCAAACAAAAACUACtt	GUAGUUUUUGUUUGGUUGGtt
PAR-4.1	CCCUCAGGACAUGACCUUAtt	UAAGGUCAUGUCCUUGAGGGtt
PAR-4.2	CCUUCAUUAGUGGAGCUGAtt	UCAGCUCCACUAAUGAAGGtc
PAR-4.3	CGCCUCACUACUGGACUCUtt	AGAGUCCAGUAGUGAGGCGtt

### P815 cell culture

Cells were cultured with ATCC complete growth medium including DMEM with 4 mM L-glutamine, 1.5 mg/ml sodium bicarbonate, 4.5 mg/ml glucose, 10% FBS, 100 U/ml penicillin and 100 μg/ml streptomycin in 75-cm^2 ^tissue culture flasks (Falcon) at 37°C in a 5% (v/v) CO_2_, water-saturated atmosphere.

### Cell transfection

The cell transfection was performed according to the manufacturer's instruction. Briefly, P815 cells were collected 1 h before transfection. After washing, cells were resuspended in normal growth medium to a concentration of 1 × 10^5 ^cells/ml at 37°C. For preparation of the transfection solution, 3 μl of siPORT NeoFX Transfection agent was added into OPTI-MEM I medium to a total volume of 50 μl, and the solution was incubated for 10 min at room temperature. After dilution of small RNA in OPTI-MEM I medium to final concentrations of 3, 10, 30 and 100 nM, the RNA preparations were mixed with transfection solution for 10 min at room temperature. The RNA transfection mixture was then dispensed into a 12-well culture plate, 100 μl per well before 900 μl of cell suspension being added into each well. The transfected cells were cultured in the normal cell culture conditions for 24, 48 and 72 h before their total RNA being extracted as described above. Control cells were not transfected but processed following a similar procedure.

### Quantitative real-time PCR analysis of PAR mRNAs

Total RNA was isolated by using a TRIZOL reagent kit according to the manufacturer's instructions and stored at -80°C. Reverse transcription was performed by using a commercial RNA-PCR kit according to the manufacturer's instructions. Briefly, 1 μg of total RNA was reverse transcribed in 20 μl of solution containing 50 μM of oligo-d (T) as a primer, 10 mM of dNTP mixture, 100 U of AMV, 20 U of RNase inhibitor and 4 μl of 5 × AMV buffer. The mixture was incubated at 42°C for 30 min and 95°C for 3 min. After synthesizing cDNA from 5 μg of total RNA by using ExScriptTM RT reagent kit, quantitative PAR-1, PAR-2 and PAR-4 mRNA expression in P815 cells was determined by real-time PCR with an ABI Prism 7700 Sequence Detection System (Perkin Elmer Applied Systems, Foster City, CA, USA) following the manufacture's protocol. Each reaction contains 12.5 μl of 2 × SYBR green Master Mix, 300 nM oligonucleotide primers (Table 2), 10 μl of the cDNA (1 in10 dilution) or plasmid DNA and water, to a total of 25 μl. The thermal cycling conditions included an initial denaturation step at 95°C for 2 min, 40 cycles at 95°C for 30 s, 60°C for 30 s, and 72°C for 30 s. The RT-PCR expression of the target gene was presented as a ratio, normalized to an endogenous reference β-action and relative to a calibrator (control untransfected cells) [23].

**Table 2:** Primer sequences for real time PCR analysis

**Primer**		**Sequence**	**Size of product (bp)**
PAR-1	forward	5'-GTTGATCGTTTCCACGGTCT-3'	225 bp
	reverse	5'-ACGCAGAGGAGGTAAGCAAA-3'	
PAR-2	forward	5'-CACCTGGCAAGAAGGCTAAG-3'	298 bp
	reverse	5'-CCCAGGGTTACTGACGCTAA-3'	
PAR-4	forward	5'-GCAGACCTTCCGATTAGCTG-3'	292 bp
	reverse	5'-AGGGCTCGGGTTTGAATAGT-3'	
β-actin	forward	5'-GCTACAGCTTCACCACCACAG-3'	288 bp
	reverse	5'-GGTCTTTACGGATGTCAACGTC-3'	

### Western blot analysis

Cells were lyzed by using Triton X-100 lysis buffer for 15 min in the presence of 2 mM sodium orthovanadate, 1 mM EDTA, 50 g/ml aprotinin, 100 M leupeptin, 1 mM Dithio-DL-Treitol (DTT) and 1 mM amino-ethyl-benzenesul-fonylfuoride hydrochloride (AEBSF). After centrifugation at 1500 g for 5 min, the supernatant was mixed with β-mercaptoethanol for electrophoresis on a 12% acrylamide reducing gel. The proteins on the gel were then transferred to Immobilon PVDF membranes. The membranes were blocked overnight at 4°C with 1% BSA in Tris-buffered saline (TBS) containing 0.05% Tween 20 before rabbit anti-mouse PARs (1:500) being added for 2 h at 37°C. After washing with TBS, the membranes were incubated with peroxidase conjugated goat anti-rabbit immunoglobulins antibody (1:2000) for 1 h at 37°C, and the membranes were developed with DakoCytomation Liquid DAB + Substrate. Not transfected cells were used as negative control.

### Flow cytometry analysis

P815 cells were pelleted by centrifugation at 800 g for 5 min, and fixed with 4% Paraformaldehyde (VWR international) for 30 min on ice before being washed twice with 0.5% BSA. The cells were resuspended in PBS and incubated with FITC-conjugated rabbit anti-mouse PAR-1, PAR-2, PAR-4 polyclonal antibody or isotype control (at a final concentration 4 μg/ml) at 37°C for 2 h. Cells were finally resuspended in PBS and analyzed on a FACS Calibur flow cytometer with CellQuest software (BD Biosciences).

### Immunofluorescence cell staining

After being fixed in 4% paraformaldehyde for 30 min, P815 cells were incubated with 1% BSA for 10 min. After washing, the cells were resuspended in PBS and incubated with FITC-conjugated rabbit anti-mouse PAR-1, PAR-2, PAR-4 polyclonal antibody or isotype control (at a final concentration 4 μg/ml) at 37°C for 2 h. Images were obtained on a Nikon EZ-C1 confocal laser scanning microscope (Japan).

### Mast cell challenge

P815 cells at a density of 1 × 10^5 ^cells/ml were transfected with 10 nM of siRNAs of PAR-1, PAR-2 or PAR-4 for 48 h at 37°C before PAR expression being examined. The transfected cells were then exposed to IL-12 (10 ng/ml), trypsin (100 ng/ml, 1 μg/ml = 42 nM), tryptase (1 μg/ml, 1 μg/ml = 7.4 nM), thrombin (3 U/ml, 1 U/ml = 6.7 nM), the agonist peptides of PARs (Table 3), as well as the reverse peptides (100 μM), respectively. At 6 or 16 h following challenge, the culture plates were centrifuged at 800 g for 5 min at 4°C and culture supernatants (1 ml per well) were collected and stored at -80°C until use. The cell pellet was resuspended in PBS and analyzed on a FACS Calibur flow cytometer and confocal laser scanning microscope. Concentrations of IL-4 and IL-6 in the supernatants were quantified using commercial ELISA kits according to the manufacturer's instructions.

**Table 3:** Agonist peptides and reverse peptides of PARs used in the study

**PAR**	**Agonist peptides**	**Reverse peptides**
PAR-1	SFLLR-NH_2_	RLLFS-NH_2_
PAR-2	SLIGKV-NH_2_	VKGILS-NH_2_
PAR-4	GYPGQV-NH_2_	VQGPYG-NH_2_

### Statistical analyses

Data are expressed as mean ± SEM for the indicated number of independently performed duplicated experiments. Statistical significance between means was analyzed by one-way ANOVA or the Student's *t *test utilizing the SPSS 13.0 version. P < 0.05 was taken as statistically significant.

## Authors' contributions

LYQ carried out most experiments, generated majority of the data, and wrote large part of the first draft of the manuscript. HYZ performed in ELISA and real-time quantitative PCR and wrote a part of the first draft of the manuscript. SDW participated in data analysis and study design. SHH designed and conducted the study, and wrote the second and final drafts of the manuscript. All authors read and approved the final manuscript.
